# CD11c^+^ microglia promote white matter repair after ischemic stroke

**DOI:** 10.1038/s41419-023-05689-0

**Published:** 2023-02-24

**Authors:** Junqiu Jia, Lili Zheng, Lei Ye, Jian Chen, Shu Shu, Siyi Xu, Xinyu Bao, Shengnan Xia, Renyuan Liu, Yun Xu, Meijuan Zhang

**Affiliations:** 1 Department of Neurology, Drum Tower Hospital, The State Key Laboratory of Pharmaceutical Biotechnology, Institute of Brain Science, Medical School, Nanjing University, Nanjing, China; 2 Department of Neurology, Nanjing Drum Tower Hospital Clinical College of Jiangsu University, Zhenjiang, China; 3 Department of Radiology, Affiliated Nanjing Drum Tower Hospital of Nanjing University Medical School, Nanjing, China; 4 Jiangsu Key Laboratory for Molecular Medicine, Medical School of Nanjing University, Nanjing, China; 5 Jiangsu Province Stroke Center for Diagnosis and Therapy, Nanjing, China; 6 Nanjing Neuropsychiatry Clinic Medical Center, Nanjing, China

**Keywords:** Stroke, Acute inflammation

## Abstract

Ischemic stroke leads to white matter damage and neurological deficits. However, the characteristics of white matter injury and repair after stroke are unclear. Additionally, the precise molecular communications between microglia and white matter repair during the stroke rehabilitation phase remain elusive. In this current study, MRI DTI scan and immunofluorescence staining were performed to trace white matter and microglia in the mouse transient middle cerebral artery occlusion (tMCAO) stroke model. We found that the most serious white matter damage was on Day 7 after the ischemic stroke, then it recovered gradually from Day 7 to Day 30. Parallel to white matter recovery, we observed that microglia centered around the damaged myelin sheath and swallowed myelin debris in the ischemic areas. Then, microglia of the ischemic hemisphere were sorted by flow cytometry for RNA sequencing and subpopulation analysis. We found that CD11c^+^ microglia increased from Day 7 to Day 30, demonstrating high phagocytotic capabilities, myelin-supportive genes, and lipid metabolism associated genes. CD11c^+^ microglia population was partly depleted by the stereotactic injecting of rAAV2/6M-taCasp3 (rAAV2/6M-CMV-DIO-taCasp3-TEVp) into CD11c-cre mice. Selective depletion of CD11c^+^ microglia disrupted white matter repair, oligodendrocyte maturation, and functional recovery after stroke by Rotarod test, Adhesive Removal test, and Morris Water Maze test. These findings suggest that spontaneous white matter repair occurs after ischemic stroke, while CD11c^+^ microglia play critical roles in this white matter restorative progress.

## Introduction

Ischemic stroke elicits robust white matter damage leading to sensorimotor impairments, vascular dementia, and emotional disorders [1–3]. White matter is composed of axonal fibers wrapped and protected by myelin sheath [4]. Clinically, most stroke patients could spontaneously enter a recovery period several days after the acute stroke event, which manifests as neurological function improvement [5]. Although plenty of stroke patients could not return to normal and remain functional disability. The potential for spontaneous improvement of function is associated with intrinsic mechanisms of axonal plasticity and regeneration [2, 6]. However, little is known about white matter regeneration after ischemic stroke.

Currently, it is widely accepted that the presence of oligodendrocyte precursor cells (OPCs) and their migratory capacity are not the only limiting factors for white matter regeneration. Because of lacking a supportive cellular environment, OPCs recruited to the injured sites could not be efficiently differentiated into mature oligodendrocytes [7, 8]. Microglia are resident inspectors and scavengers in the central nervous system. Upon brain ischemia, microglia are triggered persistently and demonstrate tremendous heterogeneity that not only potentiate brain injury but also facilitate brain repair [9, 10]. Therefore, the abrupt depletion of microglial cells inhibits ischemic remyelination [11] and dysregulates neuronal network activity [12]. Whether there is a new subpopulation of microglia that promotes long-term repair during stroke rehabilitation is unclear. Accordingly, investigations of microglia subpopulations with restorative features could guide potential therapeutic strategies in stroke rehabilitation.

Studies on brain development and multiple sclerosis pointed out that microglia might exhibit white matter regenerative properties through swallowing myelin debris [7, 13, 14] and secreting neurotrophic factors [15, 16]. For instance, the study shows that microglial triggering receptor expressed on myeloid cells-2 (TREM2) activation promotes myelin debris clearance and remyelination in multiple sclerosis [14]. However, currently, communications between microglia and white matter regeneration after ischemic stroke are still lacking.

To fill in the aforementioned gaps, we performed bulk RNA sequencing of microglia sorted from various time points in the white matter regeneration stage after stroke to comprehensively exhibit microglial repairing molecular profiles. Our findings suggest that the CD11c^+^ microglia might be the potential microglial subpopulation promoting white matter recovery.

## Results

### White matter injury recovered gradually from Day 7 to Day 30 after tMCAO

**Fig. 1:**
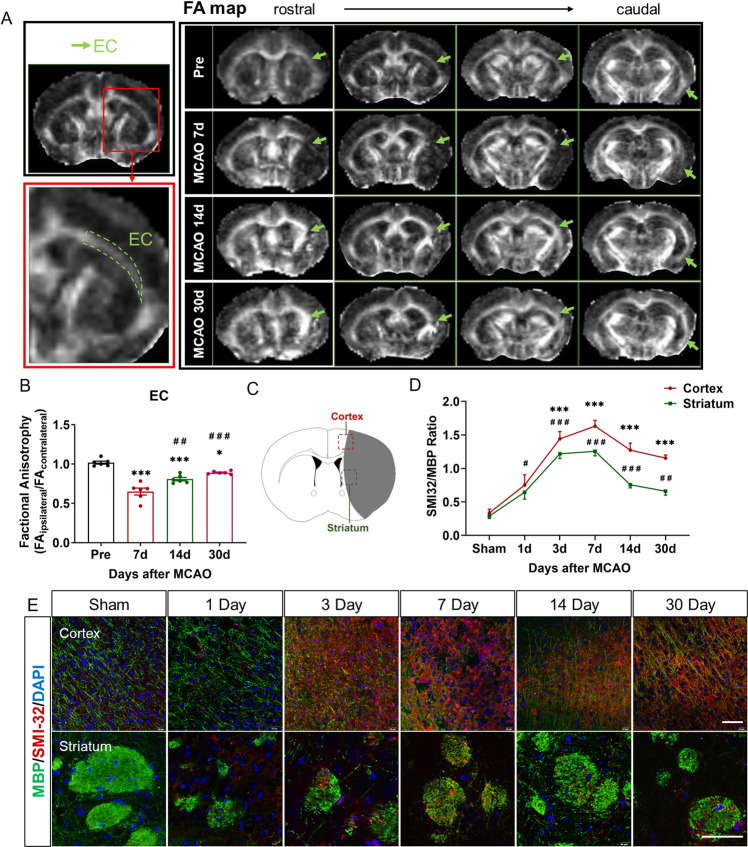
White matter injury recovered gradually from Day 7 after tMCAO. **A** Representative DTI axial views of the same mouse brain pre-tMCAO and on the 7, 14, and 30 Day post-tMCAO. Green arrowheads pointed EC. **B** Quantification of FA value expressed as the ratio of ipsilateral values to the contralateral values. *n* = 6. *p < 0.05 and ***p < 0.001 versus Pre-tMCAO group, ^##^*p* < 0.01 and ^###^*p* < 0.001 versus tMCAO 7 Day group. **C** Schematic diagram of the observation position on the cortex and the striatum of brain slices after tMCAO or sham operation. **D** The ratios of SMI32 to MBP staining intensity in the ipsilateral cortex and striatum chronologically. *n* = 6. ***p < 0.001 compared to sham group in the cortex, ^#^*p* < 0.05, ^##^*p* < 0.01, and ^###^*p* < 0.001 compared to sham group in the striatum. **E** Immunofluorescence staining of SMI32 (red) and MBP (green) in the cortex and the striatum area of sham or 1st, 3rd, 7th, 14th, 30th Day after tMCAO. Scale bars, 20 μm. For all error bars (**B**, **D**), mean values ± SEM.

To evaluate the dynamic course of white matter injury after stroke in vivo, we performed the MRI scan with DTI sequence of the same mouse before and at different time points longitudinally after tMCAO. Fractional anisotropy (FA) is a reliable parameter to quantify the white matter integrity of external capsule (EC), where is a WM-enriched area [6]. We found that the FA values significantly reduced in the EC on Day 7 post-tMCAO (0.65 ± 0.04), indicating the broken integrity of the white matter structure. The FA values of EC on the 14th (0.81 ± 0.02) and 30th (0.89 ± 0.01) day after tMCAO gradually increased but were lower than those before tMCAO (1.02 ± 0.02) (Fig. 1A). However, in the cortex and striatum, FA is not positively associated with white matter integrity [17], therefore, we performed myelin basic protein (MBP) and non-phosphorylated neurofilament H (SMI32) staining to assess white matter lesion and quantified as the ratio of SMI32/MBP values on the 1st, 3rd, 7th, 14th, 30th Day after tMCAO. The loss of white matter integrity and axonal demyelination usually presented with decreased MBP and increased SMI32 staining. The ratio of SMI32/MBP in the peri-infarct areas (Fig. 1C) of the cortex and striatum increased from Day 1 post-tMCAO and reached the highest on Day 7 (1.63 ± 0.09 in cortex and 1.26 ± 0.07 in striatum), then gradually decreased until Day 30 (1.15 ± 0.05 in cortex and 0.66 ± 0.06 in striatum), suggesting that WMI was most severe at Day 7 after tMCAO (Fig. 1D, E). Thus, the process of white matter repair could be continuous from Day 7 to Day 30 after tMCAO.

### Microglia wrapped injured white matter bundles and swallowed myelin debris

**Fig. 2:**
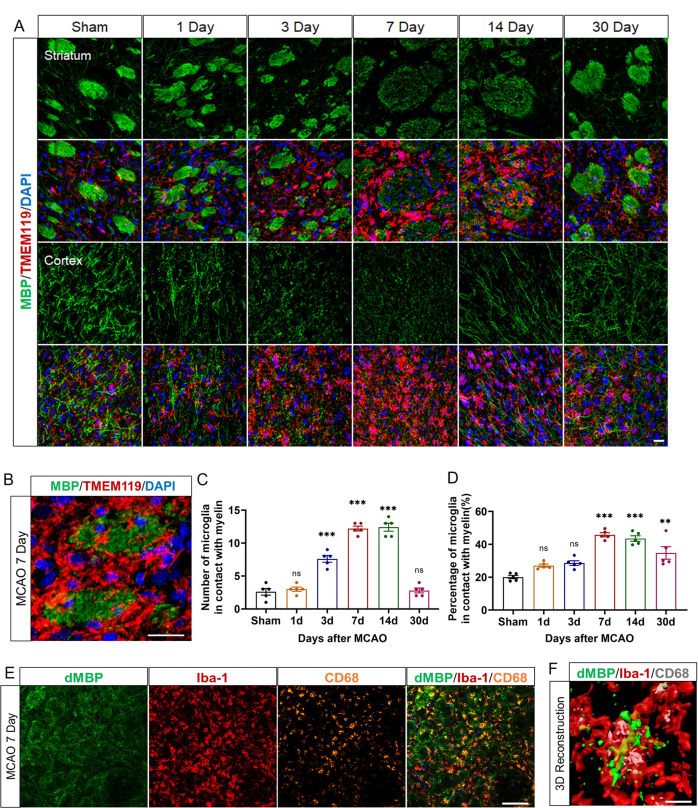
Microglia wrapped injured white matter bundles and swallowed myelin debris. **A** Representative images of MBP (green) and TMEM119 (red) immunostaining in the cortex and the striatum area of sham or 1st, 3rd, 7th, 14th, 30th Day after tMCAO mice brains. Scale bars, 20 μm. **B** Representative image of microglia centered around the damaged myelin bundles in the striatum area. Scale bar, 20 μm. **C** The number per field and **D** the percentage of microglia in contact with myelin bundles in the striatum of sham or 1st, 3rd, 7th, 14th, 30th Day after tMCAO. *n* = 5. **p < 0.01, and ***p < 0.001 compared to sham group, values were shown as mean ± SEM. **E** Representative images of dMBP (green), Iba1 (red), and CD68 (orange) immunostaining on the 7th Day after tMCAO. Scale bar, 100 μm. **F** Three-dimension reconstruction of microglia engulfed myelin debris. dMBP, green; Iba1, red; CD68, gray. Scale bar, 10 μm.

As the brain’s resident immune cells, microglia can be activated and recruited to the lesion site within hours after ischemic stroke. However, their roles in WMI after stroke are still obscure. Through dual staining of MBP and TMEM119 on the 1st, 3rd, 7th, 14th, and 30th Day after tMCAO, we observed the microglia were rapidly activated from Day 1, largely accumulated and acquired the amoeboid morphology on Day 7, and gradually turned to the resting-state on Day 30 after tMCAO in the peri-infarct areas of the cortex and striatum (Fig. 2A). Interestingly, we observed that the proliferated microglia centered around the damaged myelin bundles in the striatum area and surrounded the injured myelin sheath in the cortex (Fig. 2A). Quantitative analysis showed that the most obvious spatiotemporal microglia-myelin crosstalk happened on Day 7 and Day 14 post-tMCAO in the striatum (Fig. 2B, C). Additionally, to exclude the plausible increased contact between microglia and myelin bundles was secondary to microglia proliferation, we counted the myelin-contact microglia percentage, which also suggested the delicate communication may peak from Day 7 (45.83 ± 1.26%) to Day 14 (43.52 ± 1.69%) post-tMCAO (Fig. 2D). Using dMBP and Iba-1 to mark degraded myelin debris and microglia separately, we found that microglia engulfed myelin debris on Day 7 after tMCAO with confocal microscopy and three-dimension (3D) reconstruction (Fig. 2E, F), which showed the red semitransparent microglia wrapped in green myelin debris (Fig. 2F). Given this, we speculated that microglia communicated with damaged myelin after stroke.

### Microglia in the repairing phase of stroke exhibited molecular signatures associated with CD11c microglia population

**Fig. 3:**
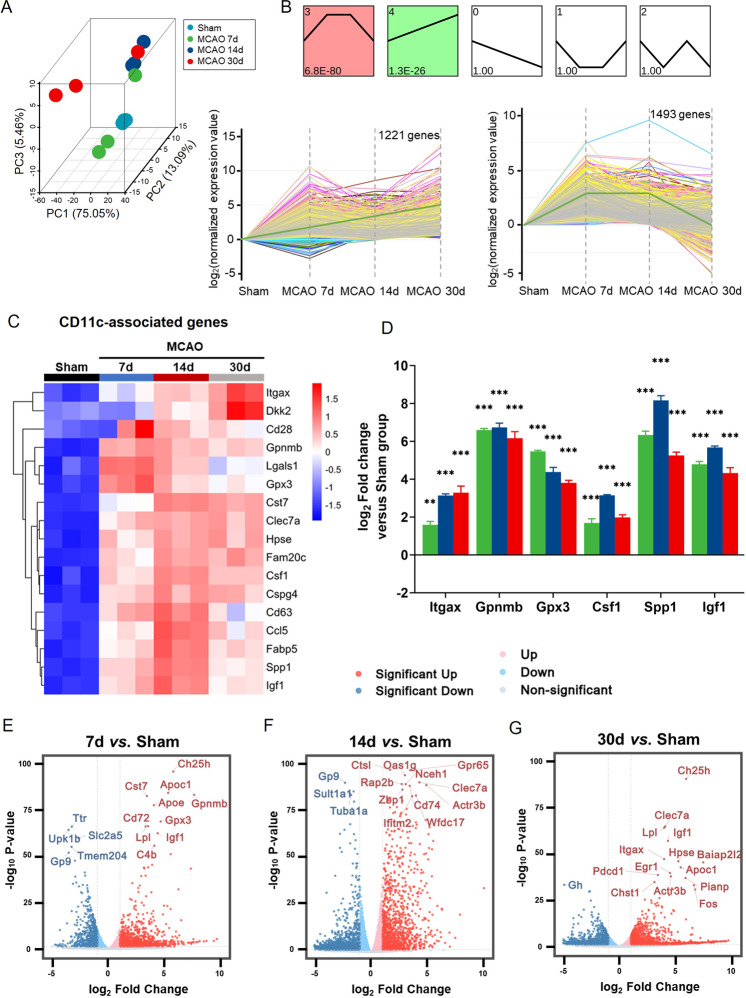
Microglia in the repairing phase of stroke exhibited molecular signatures associated with CD11c population. **A** 3D Principal Component Analysis (PCA) of the 12 samples in Sham, tMCAO 7 Day, tMCAO 14 Day, and tMCAO 30 Day groups. **B** The expression patterns of the genes were analyzed, and 5 model profiles were used to summarize the data. Each box represented an expression model profile. The upper number in the profile box represents the model profile number and the lower number indicates the *p* value. Two gene expression patterns showed significant *p* values (*p* < 0.05) (coloured boxes). Profile NO. 4 and 3 were the significant profiles including 1221 and 1493 genes, respectively. The general trend lines were green. Cluster heatmaps showing upregulation of CD11c-associated genes (**C**) on the 7–30 Day post-tMCAO. **D** qPCR for *Itgax, Gpnmb, Gpx3, Csf1, Spp1, Igf1*. *n* = 3 per group. **p < 0.01, ***p < 0.001 compared to sham group, values were mean ± SEM. Volcano plots displaying the top 15 genes according to −log_10_*p* value on the 7 (**E**), 14 (**F**), and 30 Day (**G**) versus Sham.

We observed myelin-repair and obvious spatiotemporal microglia-myelin crosstalk from Day 7 to Day 14 post-tMCAO, therefore, we supposed that Day 7 to Day 14 post-tMCAO could be the critical phase for microglia-mediated myelin repair. We then performed the transcriptional sequence of microglia sorted from the sham group and infarcted brain tissue on the 7th, 14th, and 30th-Day post-ischemia using FACS (Fig. 3A). From the total of 20,605 genes, we performed a time sequence (sham, tMCAO 7, 14, and 30 Day) profile analysis using short time-series expression miner (STEM) to search molecular dynamic alternations in line with white matter repair. Detailed STEM analysis of the microarray data presented five clusters including two significant clusters (*p* < 0.05) according to their expression patterns at different time points after tMCAO (Fig. 3B). One cluster consisting of 1221 genes was continually upregulated from Day 7 to Day 14 after tMCAO. Another cluster consisting of 1493 genes was kept at a higher level than the sham group from Day 7 to Day 14 after tMCAO. Subsequently, we performed gene ontology (GO) (Supplemental Fig. 1A) and Kyoto Encyclopedia of Genes and Genomes (KEGG) (Supplemental Fig. 1B) analysis of the interested 2714 genes. Around 14% of genes were annotated to the “immune system process,” “inflammatory response,” and “immune response” process (GO: 0002376, GO:0006954, GO:000695), while around 4.2% of genes belonged to the “Phagosome” process. We clustered phagocytosis and lipid metabolism associated genes in Supplemental Fig. 1C, D. A sub-cluster of CD11c (*Itgax*)-related genes showed a profound rise and aroused our interest.

The cluster heatmap of the CD11c-associated genes was displayed visually in Fig. 3C. Heatmaps showed the CD11c-associated genes were almost up-regulated in the myelin-repair phase. Using qPCR, we successfully validated several key genes regarding CD11c-associated molecules (*Itgax*, *Gpnmb*, *Gpx3*, *Csf1*, *Spp1*, *Igf1*) in the sham group, Day 7, Day 14, and Day 30 after ischemic stroke (Fig. 3D).

Generally, transcriptome analysis revealed 2642 differentially expressed genes (DEGs) on Day 7, 3754 DEGs on Day 14, and 2226 DEGs on Day 30 compared to the sham group (Supplemental Fig. 1E). Volcano plots showed fold changes relative to sham samples at different time points. The top 15 DEGs were marked with the gene names (Fig. 3E–G).

### CD11c^+^ microglia increased during stroke rehabilitation

**Fig. 4:**
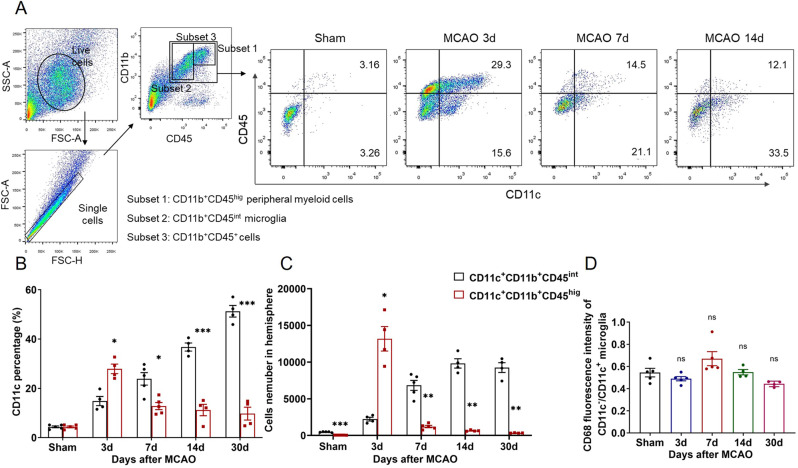
CD11c^+^ microglia continually increased from Day 7 to Day 30 after stroke. **A** Gating strategy to distinguish peripheral infiltrated myeloid cells and resident microglia (left). Representative flow cytometric analysis for the expression of CD11c in CD11b^+^CD45^+^ cells in sham, tMCAO 3, 7, and 14 Day groups. **B** Percentage and **C** cell number of CD11c^+^ peripheral cells and CD11c^+^ microglia during Subset 3 in sham, tMCAO 3, 7, 14, and 30 Day groups. *n* ≥ 4 per group. **D** The ratio of CD68 fluorescence intensity in CD11c^−^ to CD11c^+^ microglia. *n* ≥ 4 per group. **B**, **C** Compared to CD11c^+^CD11b^+^CD45^int^ group. **D** Compared to the sham group. *p < 0.05, **p < 0.01 and ***p < 0.001. Values were mean ± SEM.

In the light of RNA-sequencing, we then characterized CD11c^+^ microglia using flow cytometry. We defined mouse microglia as CD45^int^CD11b^+^, different from peripheral infiltrated myeloid cells which were CD45^hig^CD11b^+^. Gating strategy and typical images of CD11c percentage in CD45^int^CD11b^+^ population and CD45^hig^CD11b^+^ population were shown in Fig. 4A. In the intact mice, brain CD11c^+^ cells were low in both populations. Upon ischemic challenge, CD11c^+^ peripheral cells increased dramatically with the peak of 3 days after ischemia and decreased gradually thereafter. Interestingly, distinct from CD11c^+^ peripheral cells, CD11c^+^ microglia increased gradually and became the dominant CD11c^+^ population from Day 7 to Day 30 after ischemia (Fig. 4B, C). We calculated the phagocytosis capacity of CD11c^+^ microglia by measuring CD68 intensity in CD11c^+^ microglia and CD11c^-^ microglia. In all stroke time points, CD68 intensity in CD11c^-^ microglia was only half of that in CD11c^+^ microglia (Fig. 4D).

### CD11c^+^ microglia expressed higher phagocytic genes, myelin supportive genes, and lipid metabolism associated genes than CD11c^-^ microglia

**Fig. 5:**
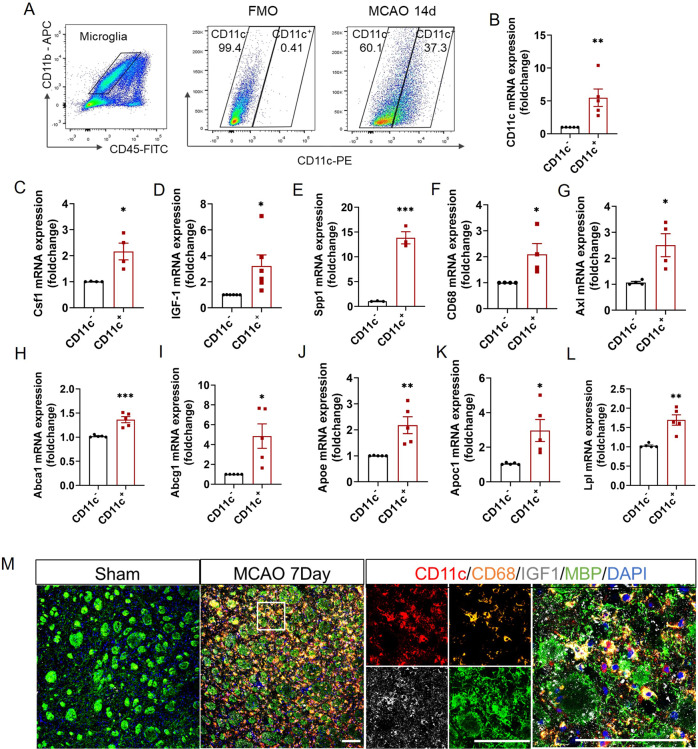
CD11c^+^ microglia expressed higher phagocytic genes, myelin supportive genes, and lipid metabolism associated genes than CD11c^−^ microglia. **A** Gating strategy and the fluorescence minus one (FMO) sample to identify CD11c^−^ and CD11c^+^ microglia on tMCAO 14d. qPCR for *CD11c* (**B**), *Csf1* (**C**), *Igf-1* (**D**), *Spp1* (**E**), *CD68* (**F**), and *Axl* (**G**) mRNA expression in CD11c^−^ and CD11c^+^ microglia, respectively. *n* ≥ 3 repeats. The results of qPCR for *Abca1* (**H**), *Abcg1* (**I**), *Apoe* (**J**), *Apoc1* (**K**), and *Lpl* (**L**) mRNA expression in CD11c^−^ and CD11c^+^ microglia, respectively, on the 7th Day post-stroke. *n* = 5 repeats. **M** Representative images of CD11c (red), CD68 (orange), IGF-1 (gray), and MBP (green) immunostaining on the sham group and 7th Day after tMCAO. Scale bars, 100 μm. *p < 0.05, **p < 0.01, and ***p < 0.001. **B**–**L** Compared to CD11c^-^ group. Values were mean ± SEM.

Then, we sorted CD11c^+^ microglia and CD11c^-^ microglia after stroke to profile their molecular characterizations (Fig. 5A). By qPCR, we found that CD11c^+^ microglia expressed significantly higher oligodendrocyte supportive genes such as *Igf1* (3.225-fold of CD11c^−^ microglia), *Spp1* (13.85-fold of CD11c^−^ microglia), and *Csf1* (2.163-fold of CD11c^-^ microglia) (Fig. 5B–E). Besides, CD11c^+^ microglia express a higher level of phagocytic associated genes (*Axl, CD68*) (Fig. 5F, G) and lipid metabolism associated genes (*Abca1*, *Abcg1*, *Apoe*, *Apoc1*, *Lpl*) (Fig. 5H–L). Immunofluorescence staining of 7th Day-post-tMCAO brain slices revealed that CD11c, CD68, and IGF-1 were multiple co-localized around the WMI area in the striatum (Fig. 5M) and in the cortex (Supplemental Fig. 2A).

### Depleting CD11c^+^ microglia exacerbated behavior performances of stroke mice

**Fig. 6:**
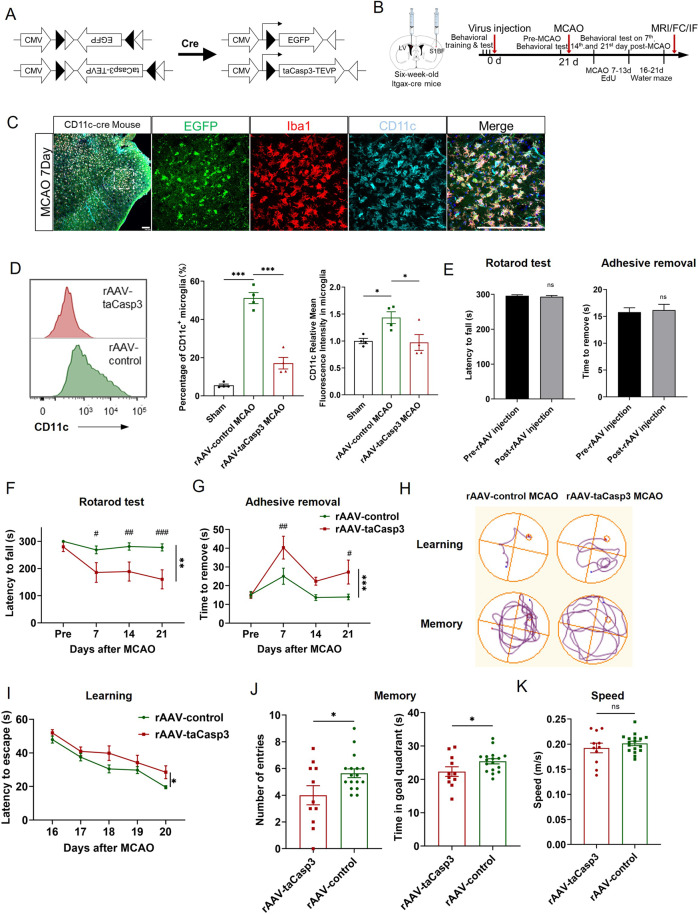
Depleting CD11c^+^ microglia in the late phase of stroke impaired functional recovery. **A** A schematic illustration of the Cre-DIO system. **B** Experimental design for CD11c^+^ depleted mice and their controls. **C** Immunostaining of CD11c-cre mice with rAAV-DIO-EGFP injection 3 weeks before tMCAO and subjected to tMCAO 7 days. Scale bars 100 μm. **D** Representative flow cytometric analysis for mice with rAAV-taCasp3 or rAAV-control injection 3 weeks before tMCAO and subjected to tMCAO 21 days. Percentage of CD11c^+^ microglia and relative CD11c fluorescence intensity of microglia in sham, rAAV-control group, and rAAV-taCasp3 group respectively (*n* = 4 per group). Stereotactic injections of rAAV did not affect the motor and sensory function of intact mice (*n* = 36). **E** Depleting CD11c^+^ microglia deteriorated long-term motor and sensory deficits after tMCAO as assessed by rotarod test (**F**) and adhesive removal (**G**) tests. *n* = 17 for rAAV-control group and *n* = 11 for rAAV-taCasp3 group. ^#^*p* < 0.05, ^##^*p* < 0.01, ^###^*p* < 0.001 compared between rAAV-control and rAAV-taCasp3 group on each day. **H** Representative images showed the swim paths in the Morris Water Maze. Cognitive functions including training test for learning (**I**), probe test for memory (**J**), and speed (**K**) were evaluated in the Morris Water Maze. *n* = 17 for rAAV-control group and *n* = 11 for rAAV-taCasp3 group. *p < 0.05, **p < 0.01, ***p < 0.001, and ns not significant. Values were mean ± SEM.

Having shown that CD11c^+^ microglia were the endogenous source of myelin-supportive genes, we then explored the functional consequence of removing CD11c^+^ microglia after stroke in vivo. CD11c-cre mice that express Cre recombinant under the control of CD11c promoter were bilateral stereotactic injected with either rAAV2/6M-DIO-taCasp3 (rAAV-taCasp3) or rAAV2/6M-DIO-EGFP (rAAV-control), respectively (Fig. 6A). Behavior tests, AAV injection, and pathological detection schemes were summarized in Fig. 6B. As shown in Fig. 6C, the EGFP^+^ cells were co-labeled with CD11c^+^ Iba1^+^ microglia on the 7th Day post-tMCAO in the infarcted area, suggesting that CD11c^+^ microglia were well-infected by rAAV2/6 M after stroke. The injection of rAAV-taCasp3 resulted in a significant reduction of CD11c^+^ microglia (17.1 ± 3.03%) in the ischemic brain 21 days after stroke compared to rAAV-control group (51.15 ± 2.87%) by flow cytometry (Fig. 6D). We further compared CD11c positive cells of rAAV-taCasp3 group to rAAV-control group in the infarcted area by CD11c immunostaining. We observed a significant reduction of CD11c intensity in rAAV-taCasp3 group (Supplemental Fig. 3A, B).

Depleting CD11c^+^ microglia did not impair the behavioral performance of intact mice (Fig. 6E). Sensorimotor functions measured by the Rotarod test and the Adhesive Removal test showed that persistent CD11c^+^ microglia deficiency exacerbated long-term neurological deficits, such as decreased time spent on a rotating bar (Fig. 6F) and increased time for removal of adhesive tapes from the impaired paws until 21 days after tMCAO (Fig. 6G). Spatial cognitive functions, as revealed by the Morris Water Maze test, manifested that mice with rAAV-taCasp3 injection spent more time in finding the hidden platform than mice with rAAV-control injection during the learning phase (Fig. 6H, I). On the probe test, mice with rAAV-taCasp3 injection swam fewer times across the goal area and spent less time in the goal quadrant after the platform was removed, indicating impaired memory retention (Fig. 6J). However, there was no difference in the swimming speed among different treatment groups. These results underscored the importance of endogenous CD11c^+^ microglia to functional recovery after stroke (Fig. 6K).

### Depleting CD11c^+^ microglia impaired white matter recovery in the late phase of stroke

**Fig. 7:**
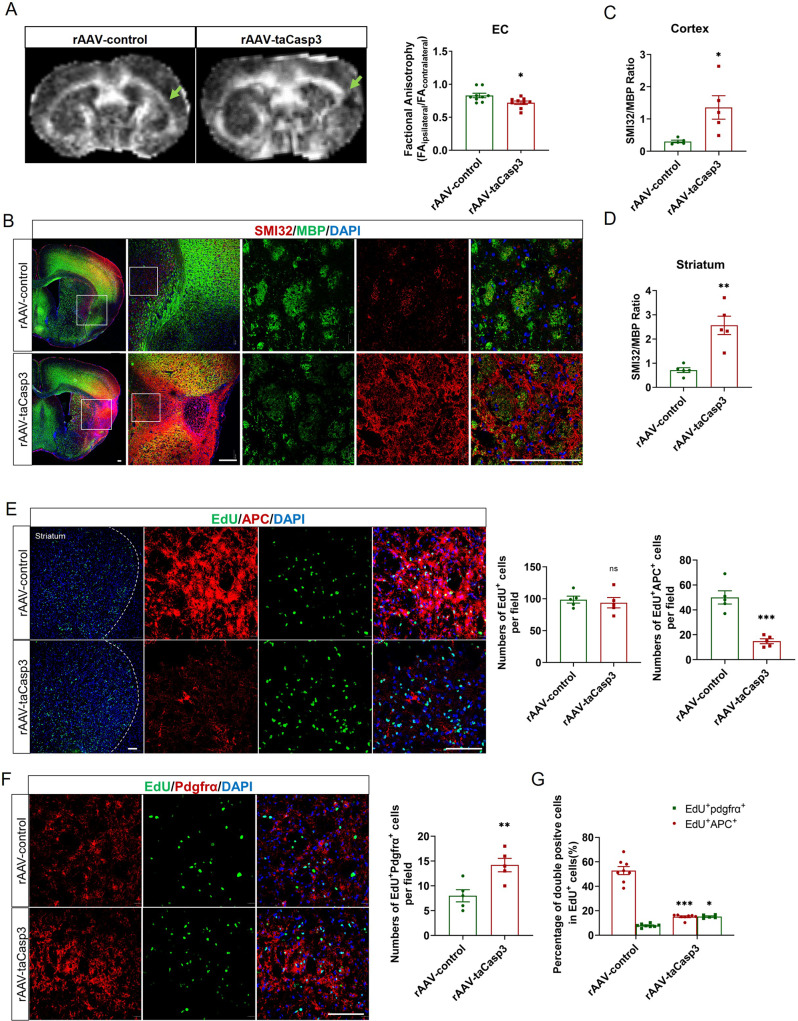
Depleting CD11c^+^ microglia in the late phase of stroke exacerbated WMI. **A** Representative DTI axial views of mice with rAAV-control and rAAV-taCasp3 injection. Quantification of FA value as the ratio of ipsilateral values to the contralateral values. *n* = 9 per group. **B** Immunofluorescence staining of SMI32 (red) and MBP (green) in the cortex and the striatum area of mice with rAAV-control and rAAV-taCasp3 injections. Scale bars, 100 μm. The ratios of SMI32 to MBP staining intensity in the injured cortex (**C**) and striatum (**D**). *n* = 5 per group. **E** Immunofluorescence staining of EdU (green) and APC (red) on the striatum area of mice with rAAV-control and rAAV-taCasp3 injections. The number of EdU single-positive cells, EdU and APC double-positive cells. *n* = 5 per group. Scale bars, 100 μm. **F** Immunofluorescence staining of EdU (green) and Pdgfrα (red) on the striatum area of mice with rAAV-control and rAAV-taCasp3 injections; EdU and Pdgfrα double-positive cells per field. *n* = 5 per group. Scale bars, 100 μm. **G** The percentage of EdU^+^ APC^+^ and EdU^+^Pdgfrα^+^ cells in EdU-positive cells. *n* = 8 per group. *p < 0.05, **p < 0.01, ***p < 0.001 compared to rAAV-control group, and ns not significant. Values were mean ± SEM.

The infarct area (detected by NeuN staining) of rAAV-control and rAAV-taCasp3 group on the 21st day after tMCAO showed no significant difference (Supplemental Fig. 4A, B). The survival rate of rAAV-control mice was similar with that in rAAV-taCasp3 mice (Supplemental Fig. 4C). We then performed an MRI DTI scan of mice three weeks after tMCAO. The DTI image showed that mice with CD11c-deleted presented a heavier WMI in the EC area, possessing a lower FA value (Fig. 7A). The dual staining of MBP and SMI32 (Fig. 7B) also confirmed that the SMI32/MBP ratio was obviously higher in the rAAV-taCasp3 mice both in the cortex (Fig. 7C) and the striatum 21 days after tMCAO (Fig. 7D). The myelin sheath detected by LFB staining showed worse white matter sheath in the external capsule of rAAV-taCasp3 group (Supplemental Fig. 5) By injecting 5-ethynyl-2’-deoxyuridine (EdU) to label newly generated cells during the myelin-repair phase from Day 7 to 14 after tMCAO, we compared the regeneration of oligodendrocytes in rAAV-taCasp3 mice and rAAV-control mice. We used APC and Pdgfrα to label mature oligodendrocytes and pre-oligodendrocytes respectively. The immunostaining images displayed that the number of EdU^+^ cells is similar between the rAAV-taCasp3 group and rAAV-control group (Fig. 7E). However, there was a significant reduction in the number and percentage of EdU^+^APC^+^ mature oligodendrocytes in the striatum in rAAV-taCasp3 mice (Fig. 7E), while the EdU^+^ Pdgfrα^+^ cell amount and percentage in rAAV-taCasp3 mice were slightly more than rAAV-control group (Fig. 7F, G). These data supported the hypothesis that CD11c^+^ microglia promote oligodendrocyte maturation and facilitate white matter repair.

## Discussion

In this current study, we found that ischemic white matter injury recovered from Day 7 to Day 30 after focal ischemic stroke. Parallel to white matter recovery, a unique CD11c^+^ microglia population increased and delivered signals necessary for white matter repair. Selective depletion of CD11c^+^ microglia using CD11c-cre mice and rAAV2/6M-DIO-taCasp3 virus diminished oligodendrocyte maturation and white matter repair after stroke. We concluded that CD11c^+^ microglia play critical roles in this white matter restorative progress.

After acute ischemic stroke, spontaneous repair of white matter damage occurs in and around the ischemic lesion [1, 18], which contributes to neurological functional recovery [18, 19]. DTI studies from rat tMCAO models demonstrated that FA values remained a continuous decrease in the ipsilateral hemisphere from 24 h to 2 weeks post-stroke and gradually recovered from the ipsilateral corpus callosum to the external capsule until 6 weeks post-stroke [6]. This FA change in corpus callosum and EC was in line with myelination and fiber density [6, 19]. Consistently, we longitudinally observed FA decline during the first week and then turned to an increase in the ipsilateral EC from Day 7 to Day 30 in the mouse focal stroke model. While in cerebral cortex, FA values were partially impacted by astrocyte gliosis [17]. Therefore, we applied MBP and SMI32 staining to trace white matter injury in cerebral cortex and striatum. In addition to EC, white matter of the striatum and cortex in peri-infarcted area also spontaneously repaired from Day 7 after ischemic stroke. In line with our results, studies from Cui et al. also reported that remyelination starts from Day 7 after tMCAO by pathological staining [20].

In an aging brain or multiple sclerosis, myelin pieces released from aging or damaged myelin sheaths could be subsequently cleared by microglia [14, 21]. However, few studies are concerned about myelin clearance after ischemic stroke. In this current study, we observed microglia centered around damaged myelin bundles after stroke and swallowed myelin debris. The spatiotemporal interaction between microglia and damaged white matter after ischemic stroke aroused our great interest in the correlation between microglia and white matter. By transcriptional analysis of sorted microglia, our study demonstrated CD11c^+^ microglia continuously expand during stroke rehabilitation until Day 30, which was in keeping with ischemic white matter recovery. In the steady state of the adult mice brains, only a small portion of CD11c^+^ cells remain. In case of brain damage or neonatal development, CD11c^+^ cells may increase and take part in immunoregulation, regeneration, and remyelination [16, 22, 23]. CD11c^+^ cells increase in the brain parenchyma of ischemic stroke models, which is a complex population derived from proliferated resident microglia and infiltrated dendritic cells (DCs) [24]. Our study demonstrated that infiltrated CD11c^+^ cells gradually decreased with the prolongation of stroke onset. However, the functional roles of CD11c^+^ microglia and their induction mechanisms during stroke rehabilitation are poorly understood. A previous study demonstrated that injecting diphtheria toxin into CD11c iDTR mice failed to knock down CD11c^+^ cells but induced a dramatic increase of CD11c^+^ cells [16]. In our study, we applied rAAV6 capsid variants with triple Y731F/Y705F/T492V mutation, which facilitated microglia infection in vivo and in vitro [25]. This construct and its based Cre-DIO system have been recently verified in microglia by several animal studies [26, 27]. Besides, peripheral infiltrated cells were scarce in the intact mouse brain. We injected rAAV2/6M virus to infect microglia before stroke, which could reduce the possible rAAV2/6M virus infection of peripheral cells. Therefore, rAAV2/6M-DIO-taCasp3 mainly affected resident microglia but not peripheral infiltrated cells after stroke. Our data demonstrated that bilateral injecting the rAAV2/6M-DIO-taCasp3 virus into CD11c-cre mice cut down more than half of CD11c^+^ microglia 21 days after tMCAO, which allowed us to verify the functional features of CD11c^+^ microglia during the late phase of stroke.

Our study demonstrated that CD11c^+^ microglia could be the dominant CD11c^+^ cells during the late phase of stroke, whose deletion exacerbated stroke mouse behavior and retarded white matter regeneration. We investigated the possible mechanisms of CD11c^+^ microglia promoting remyelination after ischemic stroke. The existence of CD11c^+^ microglia was also found to rise continually in the remote degenerative thalamus on 28 days secondary to primary tMCAO, although their functions have not been addressed [28]. Disease associated microglia (DAM) in neurodegenerative disease were CD11c positive, which participated in lipid metabolism and phagocytic pathways. DAM were considered to have the potential to restrict neurodegeneration [23, 29, 30]. Oligodendrocyte-supportive genes such as *Spp1* and *Igf1* were abundant in CD11c^+^ microglia [31]. CD11c^+^ microglia expressed the majority of IGF-1, which is necessary for myelin development. Studies in the developing mouse brain demonstrated that partial depletion of IGF-1 in CD11c^+^ microglia led to a reduction in brain weight, decrease in PLP, MBP, MAG, and MOG expression, and higher frequency of less myelinated fibers in the corpus callosum [16, 32]. In mouse early postnatal brain, one unique Spp1^+^Igf1^+^ microglia cluster residing specifically in the axon tracts of the pre-myelinated brain also expressed higher levels of lysosomal markers Lamp1 and CD68 [33]. Genes expressed in the development are often re-expressed upon brain injury. Consistently, in our study, the CD11c^+^ microglia population expressed significantly higher phagocytosis-associated genes CD68 and Axl than CD11c^-^ microglia population, indicating CD11c^+^ population may own the higher capabilities of debris clearance. Spp1 has been shown pro-myelinative effects after ischemic white matter injury [31, 34]. Colony stimulating factor 1 (Csf1), a key regulator of myeloid lineage cells, whose deletion severely impaired white matter regeneration [35]. Phagocytosis of excessive lipid-rich myelin debris may cause cholesterol overload. To keep cholesterol hemostasis, supererogatory cholesterol flows out through ABC transporters, especially ATP-binding cassette transporter A1 (ABCA1) and ATP-binding cassette transporter G1 (ABCG1). Cholesterol is insoluble in water, whose transportation requires apolipoproteins including apolipoprotein E (APOE) and apolipoprotein C1 (APOC1) [36, 37]. Myelin-clearance by phagocytes might lead to compensatory increase in the expression of lipid transporters through cholesterol derivates, which could be beneficial for cholesterol recycling and remyelination [38, 39]. Take a whole, we predicted that CD11c^+^ microglia owing swallowing capabilities and expressing higher myelin-supportive genes and lipid metabolism associated genes, which accelerated white matter repair after ischemic stroke.

To be summarized, we showed that the microglial gene expression pattern in the late phase of stroke showed a dramatic repairing signature. CD11c^+^ microglia population expressed a characteristic myelinogenetic gene profile, equipping them to play a fundamental role in white matter repair in the stroke rehabilitation stage.

## Methods

### Experimental animals

Male C57BL/6J mice and H11-Itgax-iCre mice were purchased from the Animal Model Center of Nanjing Medical University and GemPharmatech Co., Ltd. (Nanjing, Jiangsu, China) respectively. The mice were bred and housed in an air-conditioned, temperature-controlled, and humidity-controlled room under a 12-h light/dark cycle. For animal studies, there was no sample-size estimation to ensure adequate power to detect a pre-specified effect size. All animal experiments were conducted under the guidelines of the Animal Use and Care Committee of Nanjing University.

### Transient middle cerebral artery occlusion model (tMCAO) and treatment

The tMCAO model was established to mimic unilateral focal cerebral ischemic stroke as described previously [40]. After being anesthetized, a midline neck incision was made under a dissecting microscope, and then the right common carotid artery and external carotid artery (ECA) were isolated. A suture (Doccol Corporation, MA, USA) was introduced into a wedge-shaped incision on the ECA and further inserted to obstruct the middle cerebral artery. After one hour of occlusion, the filament was withdrawn for reperfusion. Cerebral blood flow (CBF) was evaluated by a Doppler laser. Mice would be excluded from the study if their relative CBF failed to reduce to 20% of baseline or showed no neurologic deficits after anesthesia recovery. Sham-treated mice were subjected to the same procedure without tMCAO. During the surgery, the body temperature of mice was maintained at 37 ± 0.5 °C with a heating pad.

### Magnetic resonance imaging (MRI)

Mice before and after tMCAO surgery received an MRI scan on a 9.4 T Bruker MR system (BioSpec 94/20 USR, Bruker) with a 440-mT/m gradient set, an 86-mm volume transit RF coil, and a single channel surface head coil. After anesthetizing with 2.5–3% isoflurane inhalation, mice were restrained on a mouse holder with tooth bars and ear bars for data acquisition and physiological parameter monitoring. Diffusion-weighted images (DWI) were acquired with spin-echo echo-planar imaging (SE-EPI) sequence with the following parameters: Two *b*-values (*b* = 0 and 1000 s/mm^2^) along with 30 non-collinear directions, *δ* = 4.1 ms, Δ = 10.3 ms; TR: 1500 ms, TE: 23.27 ms, FOV: 20 mm × 20 mm, matrix: 128 × 128, and 22 adjacent slices of 0.7 mm slice thickness. Imaging data were converted into NIFTI format with MRIcron. Diffusion data were post-processing using FSL (v.5.0.9) pipeline including corrections for eddy currents and movement artifacts (eddy_correct), rotations of gradient directions according to eddy currents corrections (fdt_rotate_bvecs), brain mask extractions based on b0 images (bet) and FA maps calculations by fitting a diffusion tensor model at each voxel (dtifit). EC areas were drawn using the itk-SNAP to extract the FA values.

### Microglia isolation from mice and flow cytometry

After sham or tMCAO surgery, mice were killed by cervical dislocation at certain time points. The right hemisphere of the tMCAO brains or sham brains, excluding brain stem and olfactory bulbs, were cut into pieces with tissue scissors and then put in a glass homogenizer for homogenizing in PMG media. Single-cell suspensions were prepared using 70μm cell filters. After centrifuging and discarding the supernatant, the cell compounds were resuspended in 30% (v/v) isotonic Percoll, overlaid with 70% (v/v) isotonic Percoll to the bottom of the solution, and centrifuged at 2000 rpm at 4 °C for 30 min with slow acceleration and no brake. The microglia-enriched cell population isolated from the 30–70% interphase was diluted 1:5 in ice-cold PBS and recovered by cold centrifugation at 1500 rpm for 5 min in microcentrifuge tubes. The obtained cells were incubated with a mixture of antibodies against CD11b-APC (1:300, eBioscience, #17-0112-82), CD45-FITC (1:300, eBioscience, #56-0451-82), and CD11c-PE (1:300, eBioscience, #12-0114-81) at 4 °C for 30 min, and CD11b + CD45int cells were sorted by BD FACSAria™ III (San Jose, CA, USA) as microglia. Appropriate CD11c isotype control (Armenian Hamster IgG Isotype Control, PE, 1:300, eBioscience, #12-4888-81) was used according to divide CD11c^-^/CD11c^+^ cells. For flow cytometry analysis, following antibodies were used: CD45, CD11b, CD11c, and CD68 (1:300, Biolegend, #333809). Appropriate isotype controls were used according to the manufacturer’s instructions (Thermo Fisher eBioscience).

### RNA-seq library construction, RNA sequencing, and bioinformatic analysis

The transcriptome sequencing and analysis were conducted by OE Biotech Co., Ltd. (Shanghai, China). Briefly, total RNA from FACS-sorted microglia was extracted using the mirVana miRNA Isolation Kit (Ambion, USA) following the manufacturer’s protocol. The samples with RNA Integrity Number (RIN) ≥ 7 were subjected to the subsequent analysis. The libraries were constructed using TruSeq Stranded mRNA LTSample Prep Kit (Illumina, San Diego, CA, USA) according to the manufacturer’s instructions. Then these libraries were sequenced on the Illumina sequencing platform (Illumina HiSeq X Ten) and 150 bp paired-end reads were generated. Raw data (raw reads) were processed using Trimmomatic. The reads containing ploy-N and the low-quality reads were removed to obtain clean reads. Then the clean reads were mapped to the reference genome using hisat2. FPKM value of each gene was calculated using cufflinks, and the read counts of each gene were obtained by htseq-count. DEGs were identified using the DESeq (2012) R package functions estimate Size Factors and nbinom Test. *p* value < 0.05 and fold change >2 were set as the threshold for significantly differential expression. Hierarchical cluster analysis of DEGs was performed to explore gene expression patterns. The software for Short Time-series Expression Miner (STEM) was provided by OE Biotech Co., Ltd., and *p* value < 0.05 was significant. GO enrichment and KEGG pathway enrichment analysis were respectively performed using R based on the hypergeometric distribution.

### Immunofluorescence staining and confocal microscopy analysis

Experimental mice were anesthetized and then transcardially perfused with PBS followed by 4% paraformaldehyde (PFA). The brains were quickly dissected, then fixed in 4% PFA overnight, and dehydrated in gradient sucrose solutions. After a quick freeze and fixation, brains were sectioned at the thickness of 20 μm for subsequent immunofluorescence staining. Brain sections were permeabilized with 0.25% Triton X-100 for 20 min followed by blocking with 2% bovine serum albumin (BSA) for 90 min and then incubated with primary antibodies overnight at 4 °C. The following primary antibodies were used: Anti-APC, 1:100, Calbiochem, #OP80; Anti-CD11c, 1:500, eBioscience, #14-0114-82; Anti-CD68, 1:500, Abcam, #ab53444; Anti-dMBP, 1:500, Millipore Sigma, #AB5864; Anti-Iba1, 1:500, Abcam, #ab178847; Anti-IGF1, 1:100, Novus Biologicals, #NBP2-34361, Anti-MBP, 1:500, Abcam, #ab7349, #ab40390; Anti-NeuN Antibody, 1:500, Abcam, #ab177487; Anti-NF-H Antibody (SMI32), 1:500, BioLegend, #801702; Anti-Pdgfrα Antibody, 1:300, BD Biosciences, #559774; Anti-TMEM119, 1:500, Synaptic Systems, #400011. The brain sections were incubated with secondary antibodies for 2 h at room temperature in the dark the next day. The nuclei were counterstained with DAPI (5 mg/ml, Bioworld Biotechnology) for 15 min. Confocal fluorescence microscopy (Olympus FV3000, Japan) was used to capture images. Image analysis was performed on three randomly selected microscopic fields of each animal. All images were captured using the same microscope settings and processed with the same parameters. The exported images were loaded into ImageJ (NIH) and were quantified by two independent observers blinded to groups. The image processing software Imaris was used to reconstruct three-dimensional images.

### Total RNA extraction and real-time quantitative PCR analysis

Total RNA was extracted from microglia sorted by FACS using the Trizol reagent (Invitrogen) according to the manufacturer’s protocol, then reverse-transcribed into cDNA with a PrimeScript RT Reagent Kit (Takara, Japan). Real-time quantitative PCR (qPCR) of the cDNA was performed using a Step One Plus PCR system (Applied Biosystems, Foster City, CA, USA) with an SYBR Green Kit (Applied Biosystems). Relative quantified levels were compared using the ∆∆Ct method normalized to the endogenous control Gapdh. The primer sequences were as follows:

Abca1 Forward CTGTTTCCCCCAACTTCTG

Reverse TCTGCTCCATCTCTGCTTTC

Abcg1 Forward TCTTTGATGAGCCCACCAGT

Reverse GGGCCAGTCCTTTCATCA

Apoc1 Forward TCCTGTCCTGATTGTGGTCGT

Reverse CCAAAGTGTTCCCAAACTCCTT

Apoe Forward CTGACAGGATGCCTAGCCG

Reverse CGCAGGTAATCCCAGAAGC

Axl Forward GGAACCCAGGGAATATCACAGG

Reverse AGTTCTAGGATCTGTCCATCTCG

CD68 Forward TGTCTGATCTTGCTAGGACCG

Reverse GAGAGTAACGGCCTTTTTGTGA

Csf1 Forward GTGTCAGAACACTGTAGCCAC

Reverse TCAAAGGCAATCTGGCATGAAG

Gpnmb Forward TGCCAAGCGATTTCGTGATGT

Reverse GCCACGTAATTGGTTGTGCTC

Gpx3 Forward CCTTTTAAGCAGTATGCAGGCA

Reverse CAAGCCAAATGGCCCAAGTT

Igf-1 Forward CACATCATGTCGTCTTCACACC

Reverse GGAAGCAACACTCATCCACAATG

Itgax Forward CTGGATAGCCTTTCTTCTGCTG

Reverse GCACACTGTGTCCGAACTCA

Lpl Forward GGGAGTTTGGCTCCAGAGTTT

Reverse TGTGTCTTCAGGGGTCCTTAG

Spp1 Forward CACTCCAATCGTCCCTACAGT

Reverse CTGGAAACTCCTAGACTTTGACC

Gapdh Forward AGGTCGGTGTGAACGGATTTG

Reverse TGTAGACCATGTAGTTGAGGTCA

### Stereotactic injections of recombinant adeno-associated virus (rAAV)

Stereotactic injections of rAAV were performed following previously published protocols [26, 27]. There was no randomization methods used for grouping. Briefly, male H11-Itgax-iCre mice were fixed in a stereotactic frame after being anesthetized with pentobarbital (20 mg/kg). The sterile ointment was applied to each eye to avoid corneal drying. After exposing the skull surface with a midline scalp incision, the target site was defined using stereotactic coordinates. The injection dose and coordinates relative to bregma were as follows: Lateral ventricles on left side: anterior/posterior (AP) −0.22 mm, medial/lateral (ML) 1.00 mm, dorsal/ventral (DV) −2.50 mm, 2 μl. The primary somatosensory barrel cortex (S1BF) on right side: AP 0.02 mm, ML 3.00 mm, DV −2.00 mm. The Cre-dependent virus of either rAAV-DIO-taCasp3 (rAAV-CMV-DIO-taCasp3-TEVp-WPRE-hGH polyA, AAV2/6 M, 5E + 12 vg/ml) or rAAV-DIO-EGFP (rAAV-CMV-DIO-EGFP-WPRE-hGH, AAV2/6 M, 5E + 12 vg/ml) from BrainVTA (Wuhan, China) was bilaterally delivered into the mice. Keep the glass microelectrode staying for 10 min at the end of each injection to avoid backflow. All the mice were given behavioral tests before and 3-week after rAAV microinjection. Mice with no differences in behavioral tests were included for subsequent experiments.

### Behavioral tests

Behavioral tests were performed by an individual blinded to experimental groups. The Rotarod test was performed to assess mouse balance and sensorimotor coordination. Briefly, mice were forced to run on a five-lane rotarod device (IITC Life Science). The rotating rod was placed horizontally, accelerating from 4 rpm to 40 rpm for 5 min. Before the operation, the mice were trained for 3 days at 20 rpm, 30 rpm, and 40 rpm separately in the morning and evening, with an interval of 15 min. On the test day, the mice were placed on the rod in turn, and the average time of latency to fall for three rounds of experiments was recorded. The adhesive removal test was performed to assess tactile responses and sensorimotor asymmetries. Two 2 × 3 mm adhesive tapes were applied to the forepaws. Tactile responses were measured by recording the time to remove the adhesive tape, with a maximum observation period of 120 s. Cognitive function was analyzed using the Morris Water Maze test following the previous protocol [41]. Briefly, during the learning test (Day 1 to Day 5), the time spent finding the submerged platform was recorded from four locations to place the mice into the pool and train the mice to stay on it for 30 s. The mice were allowed to stay on the platform for 30 s when they did not find it, and 60 s was recorded as its latency time. For the probe test on the 6th day, the mice were placed into the pool from two of the four locations which are on the diagonal and allowed to swim freely for 60 s without the platform. All the data were recorded and analyzed using the ANY-maze system (Stoelting, USA).

### EdU injections and staining

To label proliferating cells, animals were intraperitoneally injected with the 5-ethynyl-2’-deoxyuridine (EdU, 5 mg/kg, Invitrogen) once a day from Day 7 after tMCAO for 7 consecutive days. EdU staining followed the manufacturer’s introduction with Click-iT® EdU Imaging Kits (Invitrogen).

### Statistical analysis

Statistical analyses were performed with SPSS 18.0 software (IBM Corp, Armonk, NY, USA). All data were presented as the mean ± standard error of the mean (SEM). The difference between the two groups was analyzed using an unpaired Student’s *t* test for the data was normally distributed. The difference among multiple groups was analyzed by one-way analysis of variance (ANOVA) for the data with homogeneity of variance and followed by the Bonferroni post hoc test. Statistics involving time trends in different groups such as behavior tests and flow cytometry were analyzed with two-way ANOVA followed by the Bonferroni post hoc test. The variance is similar between groups that are being statistically compared. A statistically significant difference was established at *p* < 0.05.

## Supplementary information

reproducibility checklist

Supplemental Figures and methods

## Data Availability

The datasets used during the current study are available from the corresponding author on reasonable request. The microarray data of RNA-seq were deposited in the NCBI’s Sequence Read Archive (SRA) with the BioProject accession number: PRJNA809756.
