# Supplementary material for: CD11c+ microglia promote white matter repair after ischemic stroke
Source: Cell Death Dis. 2023 Feb 24;14(2):156. doi: 10.1038/s41419-023-05689-0 (PMC9958101; doi:10.1038/s41419-023-05689-0)
Supplement: Supplementary file 2 — Supplemental Figures and methods [file 41419_2023_5689_MOESM2_ESM.docx]

**Supplemental Figures**

Figure 1


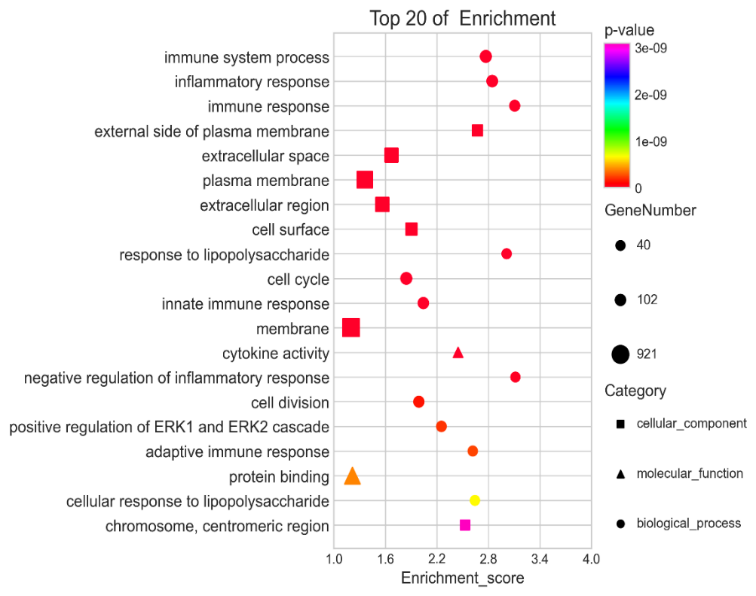

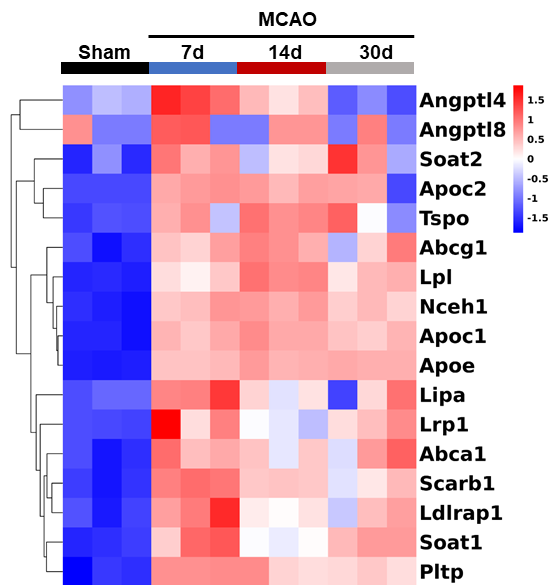

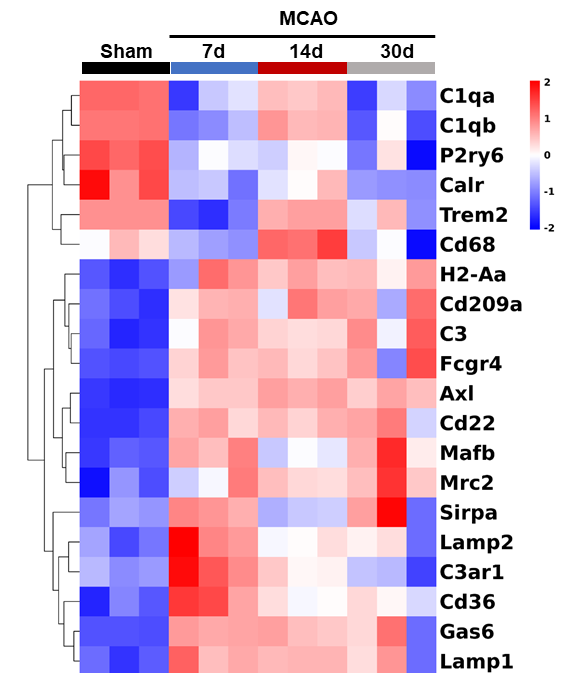

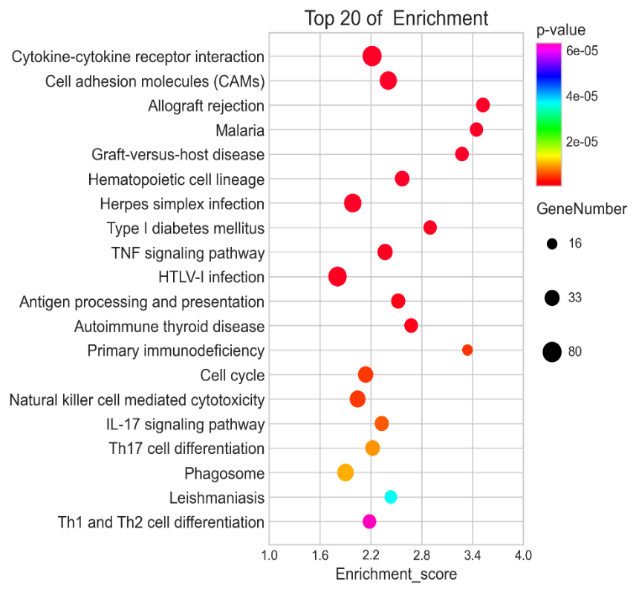


A

B

C

D

E


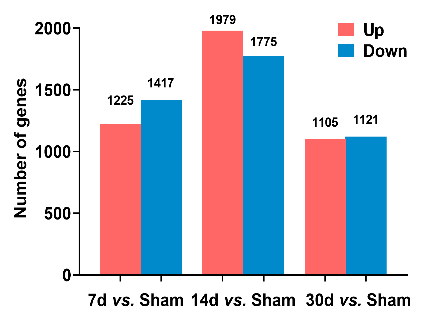


**Supplemental Figure 1.** **Transcriptional analysis of sorted microglia**

(A, B) The functional enrichment results for the interested genes were selected by STEM. GO (A) and KEGG (B) analysis of top 20 enrichment pathways are shown separately. Cluster heatmaps showing phagocytosis-associated genes (C) and lipid-associated genes (D) at different time points. (E) The number of up-regulated and down-regulated genes on the 7^th^ day, 14^th^ day and 30^th^ day post-MCAO compared with sham group (fold change>2, p<0.05).

Figure 2

A


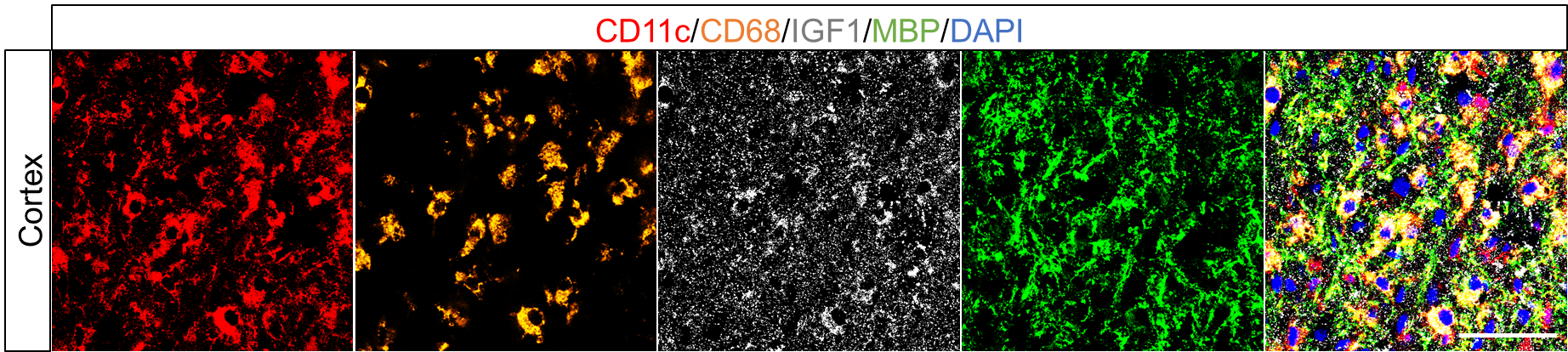


**Supplemental Figure 2. Immunofluorescence co-localization of CD11c and its-associated genes.**

(A) Representative images of CD11c (red), CD68 (orange), IGF-1 (gray), and MBP (green) immunostaining on the 7th Day after tMCAO in the cortex. Scale bars, 200 μm.

Figure 3

B

A


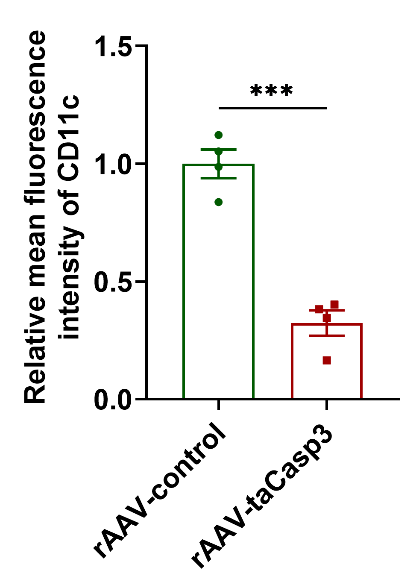

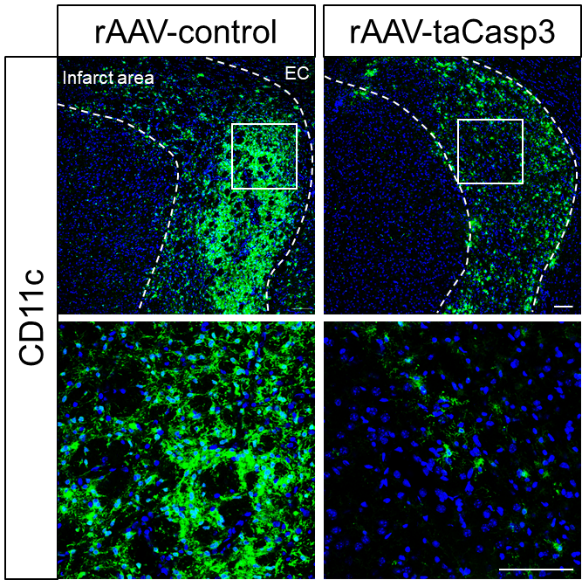


**Supplemental Figure 3. CD11c expression of rAAV-control and rAAV-taCasp3 mice on the 21st day after tMCAO.**

(A) Representative images of CD11c (green) on the rAAV-control and rAAV-taCasp3 group, 21st Day after tMCAO. Scale bars, 100 μm. (B) The relative mean fluorescence of CD11c in the infarct area in rAAV-control and rAAV-taCasp3 mice. n = 4. ***p < 0.001.

Figure 4

A


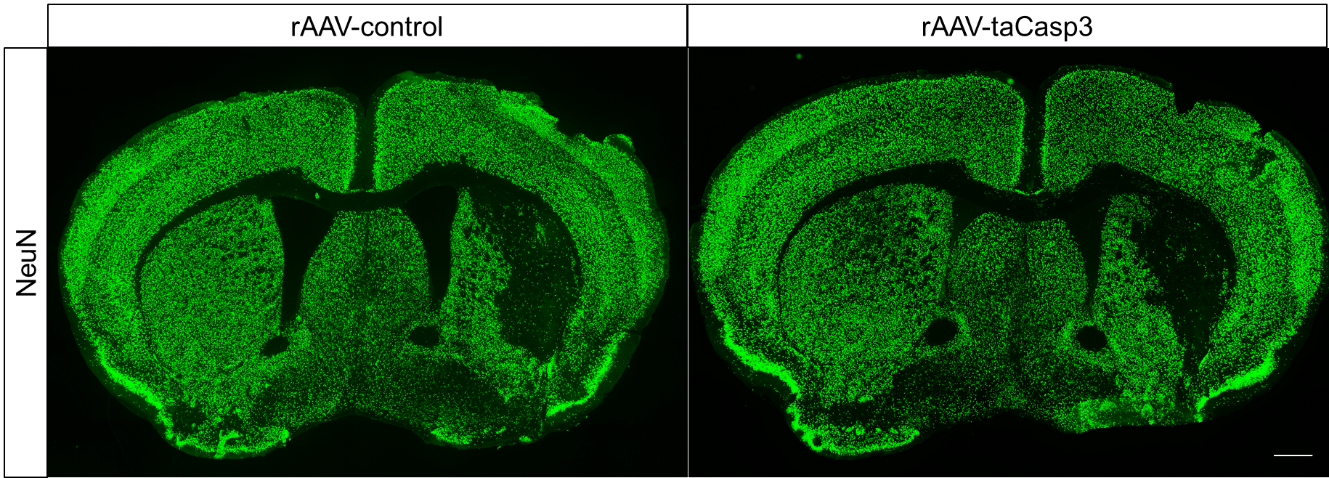


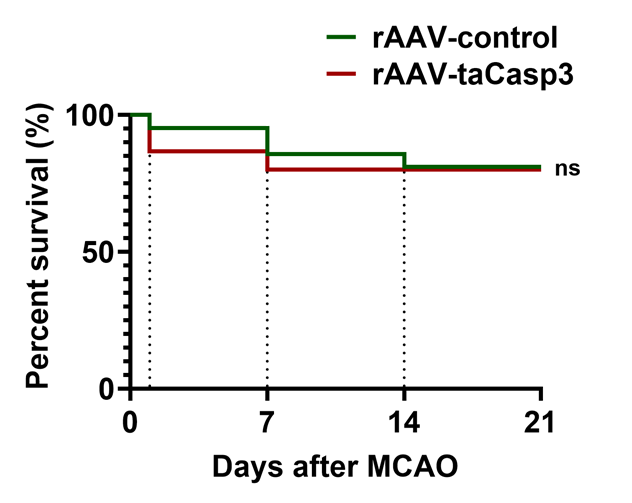

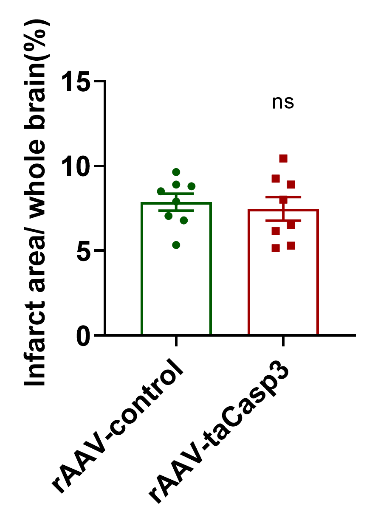


C

B

**Supplemental Figure 4. Infarct area and survival curve of** **rAAV-control and rAAV-taCasp3 mice on the 21st day after tMCAO.**

(A) Representative images of NeuN (green) in the rAAV-control group and rAAV-taCasp3 group on the 21st Day after tMCAO. Scale bars, 500 μm. (B) The percentage of infarct area in the whole brain in rAAV-control mice and rAAV-taCasp3 mice. n = 8. (C) The survival curve of the mice after MCAO in rAAV-control group and rAAV-taCasp3 group. There were 21 mice alive before tMCAO and 17 mice left on the 21st day post-tMCAO in rAAV-control group. There were 15 mice alive before MCAO and 12 mice left on the 21st day after tMCAO in rAAV-taCasp3 group. Ns indicated for no significance.

**
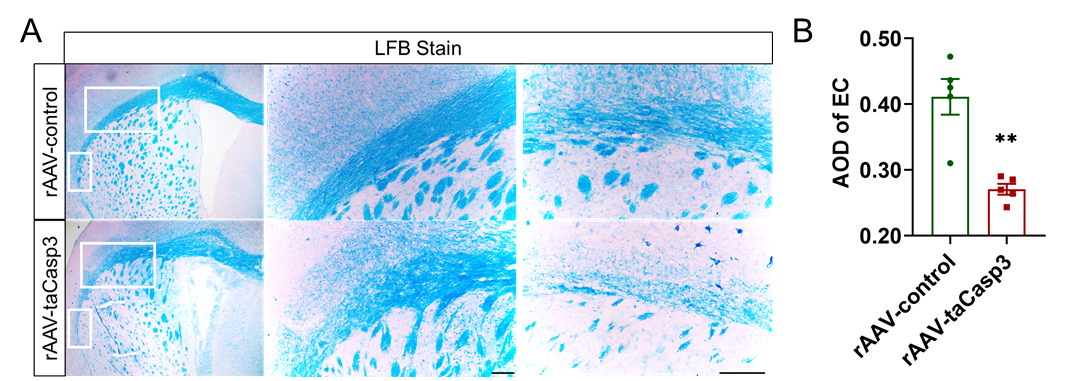
Supplemental Figure 5**

**Supplemental Figure 5 Myelin sheath of ipsilateral side 21 days after ischemic stroke detected by luxol fast blue (LFB).**

(A) Representative LFB staining images of rAAV-control and rAAV-taCasp3 group on the 21st day after tMCAO in the external capsule (EC), showing myelin bundles in the rAAV-control group were better-structured and denser than that in the rAAV-taCasp3 group. Scale bar = 100 μm. (B) Quantitative analysis of the Average Optical Density (AOD) of myelin sheath in the EC area. n = 5, **P < 0.01. Values were mean ± SEM.

**Supplemental methods：**

**Luxol fast blue (LFB) staining**

Frozen sections were stained with Luxol fast blue staining kit (Servicebio, G1030) according to the manufacturer^,^s protocol. When the myelin sheath was blue and the background was almost colorless, the slices were put into 95% ethanol and Eosin (Servicebio, G1002) counterstaining. After dehydration and sealing, the slices can be examined under microscopy (Olympus IX73) and the images were collected and analyzed.
